# Minimally Invasive Spine Surgery for Unstable Thoracolumbar Burst Fractures: A Case Series

**DOI:** 10.1055/s-0036-1594248

**Published:** 2016-11-17

**Authors:** Nitin Agarwal, Phillip A. Choi, Raymond F. Sekula

**Affiliations:** 1 Department of Neurological Surgery, University of Pittsburgh Medical Center, Pittsburgh, Pennsylvania

**Keywords:** thoracolumbar burst fracture, minimally invasive spine surgery, fusion, spine trauma

## Abstract

**Introduction** Traumatic thoracolumbar burst fracture is a common pathology without a clear consensus on best treatment approach. Minimally invasive approaches are being investigated due to potential benefits in recovery time and morbidity. We examine long-term resolution of symptoms of traumatic thoracolumbar burst fractures treated with percutaneous posterior pedicle screw fixation.

**Methods** Retrospective clinical review of seven patients with spinal trauma who presented with thoracolumbar burst fracture from July 2012 to April 2013 and were treated with percutaneous pedicle screw fixation. Electronic patient charts and radiographic imaging were reviewed for initial presentation, fracture characteristics, operative treatment, and postoperative course.

**Results** The patients had a median age of 29 years (range 18 to 57), and 57% were men. The median Thoracolumbar Injury Classification and Severity Scale score was 4 (range 2 to 9). All patients had proper screw placement and uneventful postoperative courses given the severity of their individual traumas. Five of seven patients were reached for long-term follow-up of greater than 28 months. Six of seven patients had excellent pain control and stability at their last follow-up. One patient required revision surgery for noncatastrophic hardware failure.

**Conclusion** Percutaneous pedicle screw fixation for the treatment of unstable thoracolumbar burst fracture may provide patients with durable benefits and warrants further investigation.

Burst fractures at the thoracolumbar junction due to trauma are a common pathology. 1 There is significant interest in stabilizing these patients with minimally invasive spine surgery (MIS) techniques to hasten recovery after injury. However, no real consensus has been reached in regards to the optimal treatment of such lesions. 2 3 4 5 6 7 8 9 10 11 Percutaneous pedicle screw fixation and kyphoplasty for anterior stabilization has been studied in the treatment of thoracolumbar burst fractures in both neurologically intact and impaired patients. 2 3 4 5 Results in these reports have generally been positive with significant pain reduction, kyphotic correction, and decreased perioperative morbidity and mortality compared with historical data for open surgical treatment of such lesions. 2 3 4 5 Arthrodesis has historically been used in conjunction with open or mini–open approaches to further stabilize patients. 11 We have tried to optimize outcomes by using percutaneous techniques for screw fixation, then adding fusion grafts for additional stability while maintaining gains in morbidity and recovery time associated with MIS. This case series reports long-term follow-up for patients with traumatic thoracolumbar burst fractures treated with posterior percutaneous screw fixation.

## Methods

A retrospective clinical review was conducted of adult patients with spinal trauma who presented with thoracolumbar burst fracture from July 2012 to April 2013 at a single institution and were treated with percutaneous pedicle screw fixation. Electronic patient charts and radiographic imaging were reviewed for initial presentation, fracture characteristics, operative treatment, and postoperative course. Patients were contacted for long-term follow-up to assess resolution of pain and other symptoms from their injury.

### Surgical Technique

Patients were taken to surgery the day of or one day following injury. Pedicle screws were placed one level above and one level below the level of injury unless the fixed level would be adjacent to the thoracolumbar junction. In this case, the construct was extended an additional level. All pedicle screws were placed in the same fashion. After induction of general anesthesia, preoperative somatosensory evoked potentials were obtained and the patient was turned prone on a Jackson table. The back was prepped and draped in usual sterile fashion. The fluoroscopy unit was draped, and biplanar fluoroscopy was obtained of the injury to visualize and mark the appropriate pedicles. Paramedian incisions were made at the locations of the pedicles. Meticulous hemostasis was achieved with electrocautery. A Jamshidi needle was placed under fluoroscopy, then followed with a K-needle to confirm the trajectory of the pedicle screws (K2M, Serengeti Minimally Invasive Spine Surgery System, Leesburg, Virginia). The Jamshidi needle was removed, and dilators were placed into the wound. The pedicles were then tapped and the pedicle screws were placed. Bilateral posterolateral fusion grafts of corticocancellous bone chips and demineralized bone matrix were placed adjacent to the construct. Wounds were then closed in layers as appropriate.

## Results

Seven patients with thoracolumbar burst fracture were treated with percutaneous screw fixation. The cases are summarized in Tables 1 through 2 3 4 . The median age of patients was 29 years (range 18 to 57), and 57% were men. The mechanism of injury was motor vehicle accident in four patients and fall from height in three patients. Only two patients presented with thoracolumbar spine monotrauma. All of the patients were neurologically intact on presentation except for one patient who had cauda equine syndrome. The median Thoracolumbar Injury Classification and Severity Scale score was 4 (range 2 to 9). One patient presented with multiple thoracolumbar fractures; the rest of the patients only had a single fractured vertebra. Four patients had an injury at L1, one at L2, and one at T3. The seventh patient had multiple fractures with injuries at T10, T11, and L4. The preoperative loss of height of the injured vertebrae had a median of 17.5% (range 5 to 60%). The median preoperative Cobb angle was 8 degrees (range 4 to 21 degrees). Four patients had no canal compromise, two had 5 to 10% compromise, and two had 75% compromise.

**Table 1:** Demographics of patients undergoing MIS treatment of thoracolumbar burst fracture

Patient number	Sex	Age (y)	Mechanism	Concurrent injuries	Admission/preoperative exam	TLICS score
1	F	23	Fall from second floor	Nondisplaced radius fracture, kidney lesion	Intact	4
2	F	57	MVA	Hypotension, bilateral pneumothorax, right chest and left buttocks lacerations	Intubated and moving all extremities	4
3	M	18	MVA	None	4/5 bilateral lower extremity strength, bilateral lower extremity paresthesias, decreased rectal tone, urinary retention	9
4	M	53	Fall from ladder	Bilateral intraparenchymal hemorrhages, left sixth and seventh rib fractures	Intact	3
5	M	25	Fall from roof secondary to seizure	Left upper quadrant hematoma, inferior coccyx/sacrum fracture	Intact	2
6	M	47	MVA	Right first rib fracture	Intact	2
7	F	28	MVA	None	Intact	4

Abbreviations: MIS, minimally invasive spine surgery; MVA, motor vehicle accident; TLICS, Thoracolumbar Injury Classification and Severity score.

**Table 2:** Preoperative analysis and intraoperative complications of patients undergoing MIS treatment of thoracolumbar fracture

Patient number	Type of thoracolumbar fracture	Preoperative LOH (%)	Preoperative Cobb angle (degrees)	Preoperative canal compromise (%)	Levels of instrumentation	Intraoperative complications
1	L2 burst fracture	60	10	75	T12, L1, L3, L4	None
2	L1 burst fracture	10	4	5	T11, T12, L2, L3	None
3	L1 burst fracture	40	21	75	T11, T12, L2, L3	None
4	T10 and T11 Chance fractures	10	7	10	T8, T9, T12, L1	None
	L4 burst fracture	60	15	0	L3, L5	Anterior breach of left L3 pedicle leading to hemorrhage not requiring intervention
5	L1 Chance fracture	5	8	0	T12, L1, L2	None
6	T3 burst fracture	20	7	0	T2, T4	None
7	L1 burst fracture	15	7	0	T12, L2	None

Abbreviations: LOH, loss of height; MIS, minimally invasive spine surgery.

**Table 3:** Postoperative analysis of patients undergoing MIS treatment of thoracolumbar fracture

Patient number	Postoperative LOH (%)	Postoperative Cobb angle (degrees)	Postoperative canal compromise (%)	Misplaced screws	Length of admission (d)	Last imaging follow-up after surgery (mo)	Last LOH (%)	Last Cobb angle (degrees)	Last canal compromise (%)	Hardware failure
1	25	3	10	No	6	9	25	4	10	No
2	5	2	0	No	22	None—patient had stroke at outside hospital				No
3	30	7	5	No	5	12	30	7	5	No
4	5	11	0	No	6	18	5	11	0	No
	40	11	0	No	6	18	40	12	0	No
5	5	9	0	No		10	5	9	0	No
6	5	2	0	No	6	6	5	10	0	No
7	5	2	0	No	4	9	30	7	0	Yes—L2 screw fracture requiring revision surgery; no hardware issues 1.5 y after revision surgery

Abbreviations: LOH, loss of height; MIS, minimally invasive spine surgery.

**Table 4:** Long-term outcome of patients undergoing MIS treatment of thoracolumbar fracture

Patient number	Last clinical follow-up (mo)	Clinical outcome
1	33	Pain resolved and no complaints
2	32	Some radicular pain not likely to be due to injury, but otherwise well
3	32	Complete recovery of neurologic function with no pain
4	29	Experiences some stiffness, but no pain
5	10	Some residual pain, but significantly reduced from discharge
6	6	Pain resolved and no complaints
7	38	Still in pain despite revision surgery

Abbreviation: MIS, minimally invasive spine surgery.

All patients underwent surgery using the procedure described previously. Patients were instrumented either one or two levels above and below the level of the fracture at the surgeon's discretion. Only one intraoperative complication occurred: in the patient with multiple fractures, an anterior breach of the left L3 pedicle led to a hemorrhage. No intervention was required for the hemorrhage. Immediate postoperative imaging revealed no misplaced screws. The loss of height was reduced to a median of 5% (range 5 to 40%), and the Cobb angle was corrected to a median of 5 degrees (range 2 to 11 degrees). All four patients with preoperative canal compromise had significant resolution of the canal compromise. The median length of admission for our patients was 6 days (range 4 to 22). All radiographic factors remained stable at the last imaging follow-up (median 9.5 months, range 6 to 18 months).

**Fig. 1:**
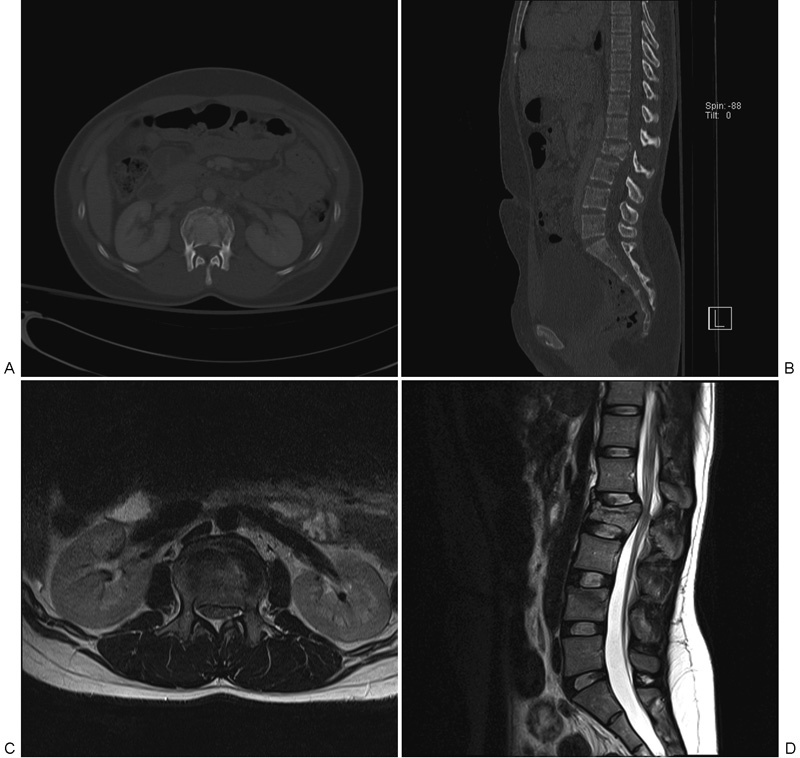
Preoperative assessment of lumbar spine. Burst fracture is noted at L2 vertebra. (A) Axial computed tomography (CT). (B) Sagittal CT. (C) Axial T2-weighted magnetic resonance imaging (MRI). (D) Sagittal T2-weighted MRI.

**Fig. 2:**
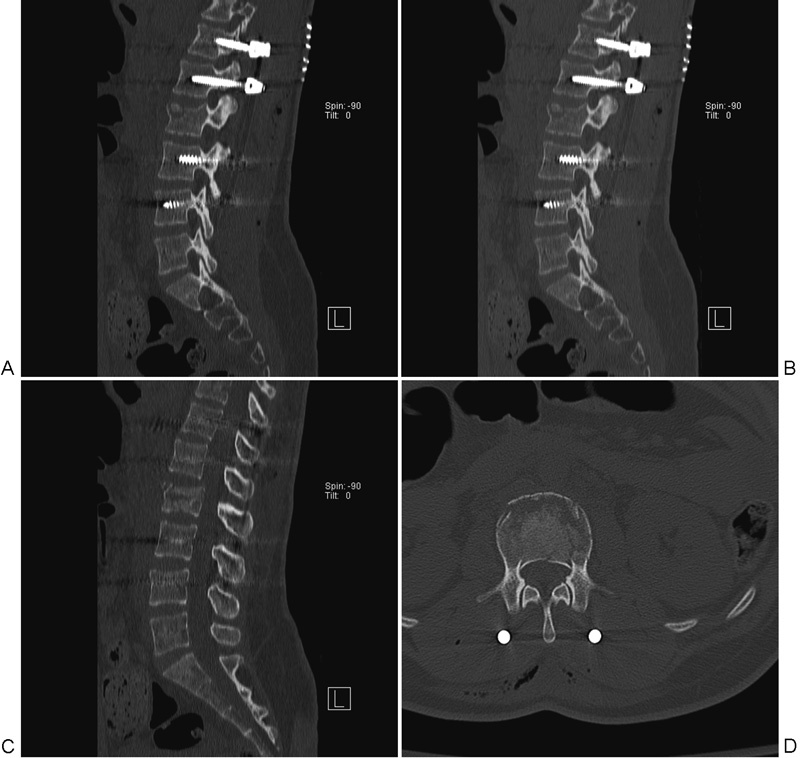
Postoperative computed tomography assessment of lumbar spine. Significant improvement of L2 vertebra loss of height, Cobb angle, and canal compromise is noted. (A) Sagittal view of left-side screw placement. (B) Sagittal view of right-side screw placement. (C) Midline sagittal view. (D) Axial view.

Five of the seven patients were reached via telephone to assess long-term resolution of pain and other symptoms. On average, the follow-up was 32.8 months (range 29 to 38) for these five patients. The last follow-up of the two patients who could not be reached via telephone was at the time of their last imaging follow-up at 10 and 6 months. Six of the seven patients had near-complete to complete resolution of their lower back pain. Figs. 1 to 2 3 4 demonstrate radiographic findings for patient number 1, who had an excellent outcome.

**Fig. 3:**
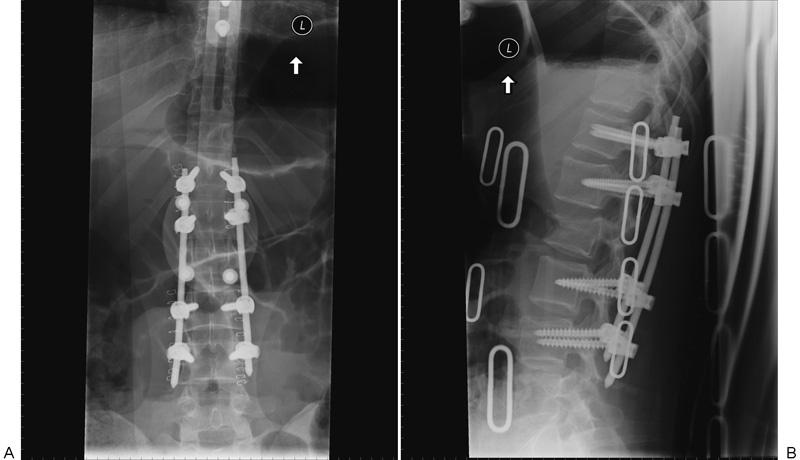
Postoperative plain film assessment of screw placement. Excellent pedicle screw placement is seen. (A) Anteroposterior view. (B) Lateral view.

**Fig. 4:**
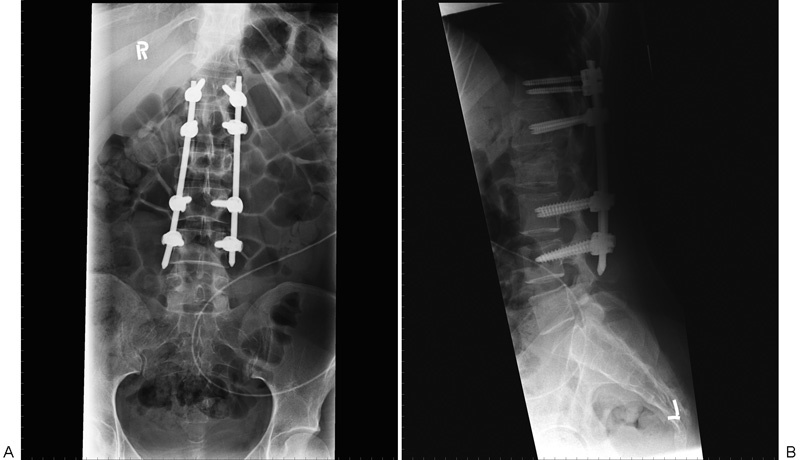
Plain film at 9-month follow-up of T12–L2 construct. Preservation of deformity correction and no migration of pedicle screws are demonstrated. (A) Anteroposterior view. (B) Lateral view.

The patient with cauda equina syndrome had complete recovery of neurologic function without pain at last clinical follow-up. The one patient who required an open revision is still in significant pain and has been working closely with a pain specialist to control her symptoms.

## Discussion

Currently, there is no consensus on the optimal treatment of thoracolumbar burst fractures. Due to improvements in spinal instrumentation technology and improved operator skill, multiple groups have argued for the use of percutaneous pedicle screw placement as part of the treatment for this pathology. As noted by Dhall et al in 2014, percutaneous pedicle screw fixation for unstable thoracolumbar fusion has not been examined in the literature with sufficiently long follow-up. 11

Many studies have examined the use of percutaneous pedicle screw placement for thoracolumbar burst fractures. Shen et al compared posterior fixation versus nonoperative treatment and found no difference in functional outcome at 2 years, but short-segment fixation showed advantages in pain relief and kyphosis correction. 12 Blondel et al treated 29 patient with A3 fractures using a combination of percutaneous pedicle screw placement and balloon kyphoplasty and achieved 11 degrees of local kyphosis correction with a 2-degree angle loss at last follow-up of 24 months. 2 No cases of screw migration were noted; however, two patients had lateral cement leakage. All patients had significant improvement in pain. This findings are similar to the series by Fuentes et al. 13 Korovessis et al treated 18 patients with percutaneous pedicle screw fixation and balloon kyphoplasty with similar results out to an average of 22 months. 3 Yang et al treated 21 patients with thoracolumbar burst fractures with percutaneous pedicle screw fixation and provided data out to 6 months postoperatively. 4 Five patients in their series required single-level decompressive laminectomies. Patients had significant kyphotic angle correction (mean correction of 6.1 degrees) and restoration of anterior vertebral height (mean difference 15.6%) that was durable at 6 months postoperatively. Ni et al treated 36 patients with single-level thoracolumbar burst fractures with percutaneous pedicle screw fixation with results similar to Yang et al with an average of 48.5 months follow-up. 5 Dai et al treated 73 patients with thoracolumbar burst fractures using posterior short-segment fixation and found no difference with or without fusion. 14

Our series includes longer-term follow-up than most of the aforementioned studies. Our patients generally reported good pain and functional outcomes following their surgeries. One patient required a second surgery after T12–L2 pedicle screw fixation due to increased back pain resulting from a left L2 screw fracture, increased anterior wedging at L1, and focal kyphosis at T12–L1. The length of stay for our patients is longer than some in the literature, but this is likely due to the other traumatic injuries sustained by our patients in addition to their thoracolumbar injury.

Several studies have investigated the use of balloon kyphoplasty for management of thoracolumbar burst fractures. One benefit of posterior fixation is the avoidance of risks associated with balloon kyphoplasty. In the Blondel et al and Korovessis et al series, 2 of 29 and 4 of 18 patients, respectively, had bone cement leakage without clinical sequelae. 2 3 A recent report by Hübschle et al examined 625 patients with 819 vertebrae treated with balloon kyphoplasty and found complication rates of 22.1 and 15.3% for cement extrusion with extrusion into intervertebral spaces and without extrusion into intervertebral spaces, respectively. 15 Five patients suffered from radiculopathy secondary to cement extrusion. Meta-analyses have reported rates of symptomatic cement extrusion to be between 0.2 and 1.5%. 16 17 18 19 20 21 In comparison, one intraoperative complication was noted within our series, yielding a rate of 14.3%. Further study with a larger sample size is necessary to ensure validity.

Postoperatively, six of the seven patients within our series had good outcomes during their follow-up. However, one patient with multiyear follow-up experienced hardware failure after 8 months of significant pain relief. Revision surgery provided stabilization for 38 months (with ongoing follow-up at this time), although she developed lower back and bilateral thigh pain after several months of pain relief when she became pregnant. This pain was attributed by a pain specialist to bilateral sacroiliac joint dysfunction, which was found in a prospective study to have an incidence rate of 6.3% in pregnant women. 22 The pain was treated with joint injections and oral medications. It is unclear what role her hardware failure and subsequent revision surgery played in the development of sacroiliac joint dysfunction. However, this case highlights the need for long-term follow-up with these patients given that hardware failure can occur many months after surgery. Currently, the literature reports pedicle screw fracture to be a not uncommon event. In a report of 38 patient with pedicle screw instrumentation for thoracolumbar burst fractures, Carl et al described two cases of broken pedicle screws and seven cases of bent pedicle screws during an average follow-up of 22 months. 23 In a study examining the effect of transpedicular grafting on short-segment pedicle screw fixation for thoracolumbar burst fractures, Alanay et al reported 2 of 20 patients with pedicle screw fracture. 24 Further investigation is required to determine if the risk of pedicle screw fracture, or other hardware failure, is significantly different in our procedure from accepted values in the literature due to a seven-patient series.

Ultimately, treatment of unstable thoracolumbar burst fractures with percutaneous pedicle screw fixation may be a viable strategy that requires further investigation to evaluate long-term outcomes and adverse effects in large cohorts. This investigation provides some evidence that such studies will be warranted due to the resolution of pain seen in the majority of our patients.
